# *Islet1* and Its Co-Factor *Ldb1* Are Expressed in Quiescent Cells of Mouse Intestinal Epithelium

**DOI:** 10.1371/journal.pone.0095256

**Published:** 2014-04-22

**Authors:** Evgeny Makarev, Marat Gorivodsky

**Affiliations:** Section on Mammalian Molecular Genetics, Laboratory of Mammalian Genes and Development, Eunice Kennedy Shriver National Institute of Child Health and Human Development, Bethesda, Maryland, United States of America; Baylor College of Medicine, United States of America

## Abstract

*Islet1* belongs to Lim homeobox (*Lhx*) gene family which encodes transcription factors that have been conserved in evolution. They form complexes with other transcriptional regulators, among them obligatory co-factors encoded by *Ldb* genes. *Isl1 (Islet1)*, *Lhx* and *Ldb1* genes play a crucial role in organ patterning, cell fate determination and cell differentiation in both embryonic and adult tissues. In this study we analyzed expression pattern of *Isl1* and its co-factor *Ldb1* in small intestine. We also studied the biological role of *Ldb1* in gut endoderm. Quantitative PCR analysis revealed a relatively high level of expression of *Lhx1, Isl1, Isl2, Lmx1a, Ldb1* and *Ldb2* mRNAs in the gut tissue as compared to the level of less abundant detectable *Lmx1b* mRNA. Immunohistochemical studies demonstrated a unique pattern of Ldb1 and Islet1 proteins in the crypt compartment. Ldb1 is produced at a low level in majority of crypt cells; but, its abundant expression was demonstrated for some single cells. Islet1 is also expressed in single cells of the crypt. Double staining experiments with Ldb1 and Isl1 antibodies showed that both genes are co-expressed in certain cells of the crypt. Further analysis revealed the Ldb1-expressing cells in the gut are both of endodermal and mesodermal origin. Proliferation studies using antibodies to phospho-histone H3 and Ki-67 antigens, as well as long-term BrdU labeling, showed that cells prominently expressing Ldb1/Islet1 are quiescent but do not belong to any known terminally differentiated cell lineages. They may represent a group of stem-like cells in the crypt. Further experiments by cell lineage tracing should be performed to better characterize this cell population. Functional studies of mice with *Ldb1* gene ablated in gut endoderm revealed no specific role of *Ldb1* in that tissue.

## Introduction

Intestinal endodermal cells represent a specific type of epithelium with relatively short lifespan. The cell turnover for all epithelial lineages in mouse small gut is less than one week. The intestinal epithelium is comprised of two separate compartments: the villus, where cells are terminally differentiated and no longer capable of dividing and the crypt, where actively proliferating cells are located [1].

There are four cell types in the gut epithelium: enterocytes, Goblet, Paneth and enteroendocrine cells. The most numerous cell populations in the intestine are enterocytes. They represent a polarized gut epithelium, and their function is to absorb nutrients. Goblet cells secrete mucin, which protects and lubricates the intestine. These cells are evenly spread throughout the villus and can be found in the crypt as well [2]. Enteroendocrine cells of the gut contain numerous neurosecretory granules and produce secreted peptide hormones. Like Goblet cells, these cells are found throughout the epithelium in both villi and crypts [2]. Paneth cells, located at the bottom of crypts, contain large secretory granules and demonstrate phagocytic activity [3]. There are also intraepithelial lymphocytes (IELs), which are intercalated in between cells of the intestinal epithelium [4].

Intestinal stem cells responsible for the constant cell renewal are localized at the bottom of the crypt [5], [6]. Each crypt contains population of stem cells and transitory population of more rapidly dividing progenitors that later migrate from the crypt to the base of the villus where they complete differentiation [5]. At the tip of the villus they undergo apoptosis and exfoliation. Several factors had been suggested as markers of intestinal stem cells and/or early progenitors including Musashi-1 [7], [8], PTEN [9], Lgr5 [10] and Bmi1 [11] [12]. The current paradigm supports the existence of two subpopulations of stem cells in crypt. The first group represents a population of small cycling cells which are marked by Lgr5 expression. Another cell population is found above the Paneth cells at position 4 and is identified as quiescent DNA label-retaining cells (LRC) [13].

Several candidates of gene markers such as *Bmi1* and *Lrig1* were proposed to be expressed specifically by LRC stem cells [12] [14]. However, the latest studies revealed that all genes previously identified as markers for LRC cells also were highly expressed by Lgr5^+^ rapidly cycling cells as well [15]. Thus LRC cells markers remain to be identified.

LIM homeodomain (Lhx) transcription factors belong to a family of Zn-finger transcription factors. They have two conserved domains: the homeodomain, which facilitates interaction with promoters of the target genes, and LIM domain, which is responsible for the protein-protein interactions (reviewed in [16]). LIM homeodomain proteins form multiprotein complexes with Ldb1 (**L**IM **d**omain **b**inding protein) and Ldb2 co-factors. Formation of these complexes was shown to substantially facilitate the activity of transcription factors [17], [18]. The role of these protein complexes in cell fate determination, differentiation and embryo patterning is well established [19]. Targeted deletion of the *Lhx1* gene leads to severe anterior embryo truncation and lack of kidney and gonad formation [20]. Its function in the patterning, cell movement and differentiation of anterior gut has been also described [21]. *Isl1* plays an important role in cell fate determination of certain interneuron populations in neural tube [22], [23]. Its transcripts were found in islet cells and mesenchymal cells of pancreatic anlagen [24]. Mice deficient in *Isl1* show complete loss of islet cells. In addition, exocrine cells of the dorsal pancreas fail to differentiate [24]. Importantly, a high level of *Isl1* mRNA has been found in the midgut of teleost fish [25].

Recent studies demonstrate the role of LIM homeodomain transcription complexes in maintenance of stem cells and/or early progenitors of several somatic tissues. *Isl1* has been shown to express in cardiac stem cells [26] and to act as a key regulator of the secondary heart field [26], [27]. Role of *Lhx2* in specification and maintenance of the hair follicle stem cells was demonstrated [28]. *Lhx2* functions downstream of signals necessary to specify hair follicle stem cells, but upstream of signals that induce final commitment. Finally, the role of *Ldb1* in development of hepatocellular carcinoma was recently described [29]. Conditional targeting of *Ldb1* gene in the liver endoderm results in a significantly enhanced growth of liver cancer and over-expression of the cyclin D1 and liver stem cell marker EpCAM.

Together these data demonstrate the role of the LIM homeodomain proteins and their co-factors in the maintenance of specific somatic stem cells and/or early progenitors as well as their ability to direct cell fate and regulate differentiation. However, the role of these transcription factors in the maintenance of the gut endoderm and/or differentiation of specific intestinal cell types remains unknown.

In this study we evaluated the expression of several LIM homeobox genes and Ldb1/Ldb2 co-factors in mouse small intestine. We found the *Lhx1, Isl1, Isl2, Lmx1a, Ldb1* and *Ldb2* mRNAs are expressed abundantly in the small intestine. Immunohistochemistry studies revealed a specific expression pattern of Isl1 and Ldb1 proteins in the intestinal epithelium. Ldb1 is expressed weakly in all cells of the crypt, while it was shown to be highly expressed in single isolated cells of the crypt. Islet1 is co-expressed with Ldb1 in few cells of the crypt, and its protein was found only in cells prominently expressing Ldb1. Probing tissue sections with antibodies to Ki67 proliferation marker and BrdU retention experiments showed that Isl1^+/^Ldb1^high^ expressing cells belong to population of quiescent intestinal cells. These data, as well as localization of Isl1/Ldb1 positive cells at the bottom of the crypt in the stem cell niche, suggests that the Isl1^+^/Ldb1^high^ could be a possible marker for the LRC stem cell population. However, functional studies in mice with targeted deletion of *Ldb1* gene in gut endoderm revealed no morphological changes in small intestine under normal physiological conditions.

## Materials and Methods

### Ethics Statement

All animals were handled humanely in accordance with the Institutional Animal Care and Use Committee (IACUC) rules and regulations at Eunice Kennedy Shriver National Institute of Child Health and Human Development. The experiments with animals used in this study were approved by the Animal Care and Use Committee of the National Institute of Child Health and Human Development (NICHD ACUC).

### Mice

The *Ldb1 ^fl/fl^* allele was described previously [30]. The *Ldb1 ^fl/fl^* mice were intercrossed with *Villin-cre* mice (Jackson Laboratory, Bar Harbor, Maine) to generate *Villin-cre Ldb1 ^fl/fl^* mouse strain. All experiments were done on two months old animals with C57/B6 genetic background. Mice were bred and maintained at the NICHD (NIH, Bethesda, MD USA) animal care facility.

### Quantitative PCR

Total RNA was isolated from mousesmall intestine by TRI-reagent (Molecular Research Center), and cDNA was synthesized using iScript cDNA syntesis kit (Bio-Rad). qPCR was performed with iQ SYBR Green Supermix (Bio-Rad) on iCycler Real Time (Bio-Rad). We used the following primers for real-time PCR: Lhx1F: AGACTGGCCTCAACATGCGTGTTA R: GTGCCAGGATGTCAGTAAATCGCT (PCR fragment 399 bp). LHX5 F: CTCATCGGACAAGGAAACCGCTAACA R: GGAGCGTAGTAGTCACTTTGGTAGT (PCR fragment 387 bp). Lhx2 F: AGCACACTTTAACCATGCCGACGT R: ATTGTCCGAAGCTGGTGGTGCTT (PCR fragment 309). Lhx9 F: ATCTGCTGGCCGTAGACAAACAGT R: TGCCAGTGCCATTGAAGTAAGGCA (PCR fragment 414 bp). Lhx3 F: ACCGACATTGGCACAGCAAGTGT R:TCGCTGCTTGGCTGTTTCGTAGT (PCR fragment 311 bp). Lhx4 F:ACTTTGTCTACCACCTGCACTGCT R:5′GGCTCCTCTTGACACTCTTGTAGA (PCR fragment 384). Lhx6 F:CTCTGGACAAGGACGAAGGTAGA R:CCTCTTGAGGTTCTCGATCA TGGT (PCR fragment size 480 bp). Lhx8 F: ATGACTTATGCTGGCATGTCCGCT R: AGTGCACTCTACAGAGGACCTTCT (PCR fragment size 297 bp). Ldbl F: 5′GAGGCACACACCATATGGTAACCA R: ATGAGCTCTCTGTGTTGCCGGAT (PCR fragment size 450 bp). Ldb2 F: 5′ACTGGAGCCAATGCAGGAACTGAT R:AAGTCTTCTTCGTCGTCCATGCCGTT (PCR fragment size 393 bp). Islet1 F:GCTCATGAAGGAGCAACTAGTGGAGA R: TTAGAGCCTGGTCCTCCTTCTGAA (PCR fragment size 342 bp). Islet2 F: ACGCGCTCATGAAAGAGCAGCTAGTA R:GAGTGCAAACTCGCTGAGTGCTTT (PCR fragment size 278 bp). Lmxla F:AAATGGTAGTGGGAATGCGGGCAT R:TTCTGAGGTTGCCAGGAAGCAGT (PCR fragment size 247 bp). Lmx1b F:AGTGTGTGTACCACTTGGGCTGTT R:AGGATGCCTTGAAAGCTCTTCGCT (PCR fragment size 315 bp).

All primers provided single band of predicted size on a gel. Relative quantification relates the PCR signal of the target transcripts to that of the least abundant detectable LIM-HD transcript (Lmx1b). Quantitative analysis was done by the Delta C(T) method.

### Histology and immunohistochemistry

Gut tissue was dissected and fixed in 4% paraformaldehyde/PBS overnight at 4°C. Tissue samples were embedded in paraffin and sectioned at 4 µm. Sections were stained with hematoxylin and eosin for histological analysis.

Sections were deparaffinized, rehydrated and permeabilized with 0.1% Triton-X-100. Endogenous biotin was blocked with Biotin Blocking System (Daco). Endogenous peroxidase activity was quenched with Peroxidase Blocking Reagent (Dako). Heat Induced Epitope Retrieval (HIER) was performed with antigen Retriever 2100 (Dako) in the Target Retrieval Solution, citrate pH 6 (Daco). The sections were washed in PBS and incubated with 5% blocking serum (Vector Laboratories) at room temperature for 1 hour followed by overnight incubation with primary antibodies at 4°C. Background Reducing Antibody Diluent (Daco) has been used for primary/secondary antibodies dilution. The following primary antibodies have been used in this study: rabbit anti-Ldb1 at 1∶4000 (whole serum kindly provided by L.-Q. Li and P. Love, NICHD, NIH), mouse anti-Islet1 at 1∶100 (clone 40.2D6, Developmental Studies Hybridoma Bank), rabbit anti-Islet1 at 1∶4000 (ab26122, Abcam), rat anti-CD45 (clone 30-F11, BD Pharmingen), rabbit anti-CD3 at 1∶200 (VP-RM01, Vector Laboratories), mouse anti-Multi-cytokeratine at 1∶50 (VP-C419,Vector Laboratories), mouse anti-BrdU at 1∶50 (clone Bu20a, Daco), rabbit anti-Chromogranin A at 1∶1000 (ab15160, Abcam), rat anti-Ki-67 at 1∶50 (clone TEC-3, Daco), rabbit anti-Phospho Histone H3 at 1∶100 (9701, Cell Signaling), and rabbit anti-Cleaved Caspase3 at 1∶500 (9661, Cell Signaling). Following incubation with the primary antibodies, sections were washed intensively in PBS/0,01%Tween and incubated with biotinylated secondary antibodies (Jackson Immuno Research). For chromogen immunohistochemistry, the signal was detected with Vectastain ABC kit (Vector Laboratories) and DAB/AEC (Daco) as a chromogen substrate. Sections then were counterstained with hematoxylin, and mounted with Aqua PolyMount (Polysciences). For immunofluorescence, sections were incubated with Streptavidin conjugated with Alexa Fluor 488/594 (Invitrogen), and nuclei were counterstained with DAPI (Vectashield, Vector Laboratories). For some experiments, additional signal enhancement was performed with TSA amplification system (Invitrogen).

### Microscopy

The confocal fluorescent imaging was performed on Zeiss LSM5 Pascal scanning confocal microscope operated by Zeiss LSM510 Software. The bright-field microscopy was done on Olympus BX60 microscope. Images were captured with Olympus Q-Color 6 camera, using QImaging software v.6.0 (QImaging Corp., Surrey, BC, Canada). All digital images were processed using Photoshop 6 software (Adobe Inc., San Jose, CA). Images of crypts were captured and analyzed using ImageJ software (http://rsbweb.nih.gov/ij/). Cells were quantified using Cell Counter plugin for the ImageJ (Kurt De Vos, University of Sheffield, UK). Cell position in the crypt was determined as described elsewhere [13], [35].

### BrdU labeling

Mice were injected i/p with 0.5 ml of BrdU (Sigma-Aldrich, St. Louis, MO) solution in PBS at 10 mg/ml. Following 40 minutes of injection mice were sacrificed and gut tissue was collected for further analysis. Long-term BrdU label retention experiments were performed according to Potten et al. [31], [32]. Mice were irradiated (8 Gy), followed by BrdU administration for 2 days. Animals were sacrificed on the 20^th^ day, guts were isolated, and BrdU incorporation was analyzed with BrdU specific antibodies (Daco). Experiments were repeated three times. Two mice were used for each experiment.

## Results and Discussion

### Expression of LIM homeodomain transcription factors and Ldb mRNAs in small intestine

**Figure 1:**
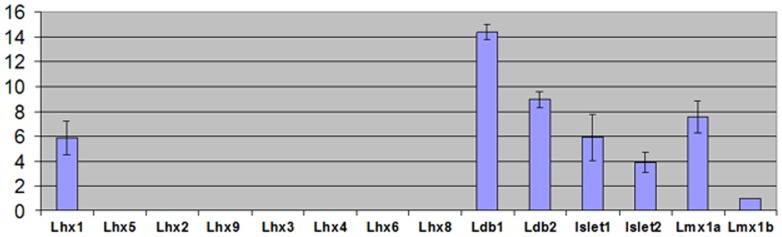
Quantitative PCR analysis of mRNA expression of the LIM homeobox genes and their cofactors. The *Lhx1*, *Isl1*, *Isl2*, *Lmx1a*, *Lmx1b*, *Ldb1* and *Ldb2* mRNAs have been detected in mouse small intestine. *Lhx1*, *Islet1*, *Ldb1* and *Ldb2* mRNAs were expressed at high level. *Lhx2*, *Lhx3*, *Lhx4*, *Lhx5*, *Lhx6* and *Lhx8* mRNAs were not detected. All values are in log2 scale and were normalized to the level of *Lmx1b* mRNA which is considered 1. At least three independent experiments were performed for each gene. The error bars show standard deviations.

To evaluate expression of LIM homeobox genes and their co-factors in mouse gut, we established qPCR screen with gene specific primers. When the level of *Lmx1b* mRNA was arbitrarily set to 1, we detected the expression of *Isl1*, *Isl2*, *Lmx1A* and *Lhx1* mRNAs at a relatively high level (Figure 1). Thus, the levels of *Lhx1* and *Islet1* mRNAs were six times higher than those of *Lmx1b* mRNA. In contrast, transcripts of *Lhx2*, *Lhx3*, *Lhx4*, *Lhx5*, *Lhx6* and *Lhx8* were not detected in mouse intestine. The highest level of mRNA expression was observed for *Ldb1* and *Ldb2* genes. The levels of *Ldb1* and *Ldb2* transcripts were more than fourteen and nine times higher, respectively, than that of *Lmx1b*. The relatively high level of expression of both *Lhx/Isl* transcription factors and their co-factors suggests a possible functional role of these transcriptional complexes in small intestine.

### Localization of Ldb1 and Isl1 proteins in crypt compartment

**Figure 2:**
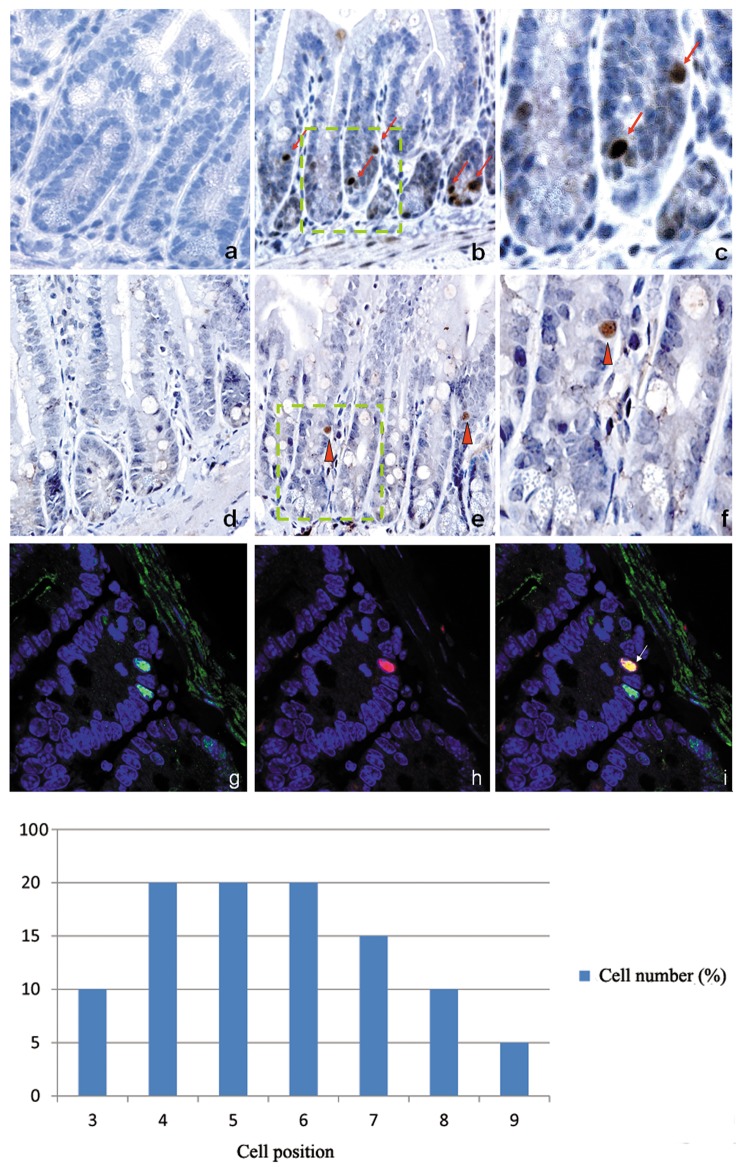
Localization of Ldb1- and Isl1-expressing cells in small intestine. Low level of Ldb1 protein is detected in most cells of the crypt (b and c). In a few single cells Ldb1 is expressed abundantly (arrows in b and c). Isl1 expression is observed in single cells of the proliferative zone of the crypt (arrowheads in e and f). No cells producing Ldb1 and Isl1 are found in the villous epithelium (data not shown). **a** and **d**. Negative control with pre-immune serum (x400). **b**. Expression of Ldb1 protein in the crypt (x400). **c**. High magnification of the selected area from **b** (x1000). Arrows point on cells which are highly expressing Ldb1. **e**. Expression of Isl1 protein in the crypt (x400). **f**. High magnification of the selected area from **e** (x1000). Arrowheads show cells which are prominently expressing Isl1. **g-i**. Co-localization of Ldb1 and Isl1 (x1000). Ldb1 (green) and Isl1 (red) are co-expressed (yellow) in single cells of the crypt. **g**. Ldb1 expression, **h**. Isl1 expression. **i**. Merge of **g** and **h**. Arrow pointed on cell which is co-expressing Ldb1 and Isl1. **j**. Location of Ldb1^high^ cells in the crypt. Ldb1^high^ cells occupied predominately position +4–+7.

**Figure 3:**
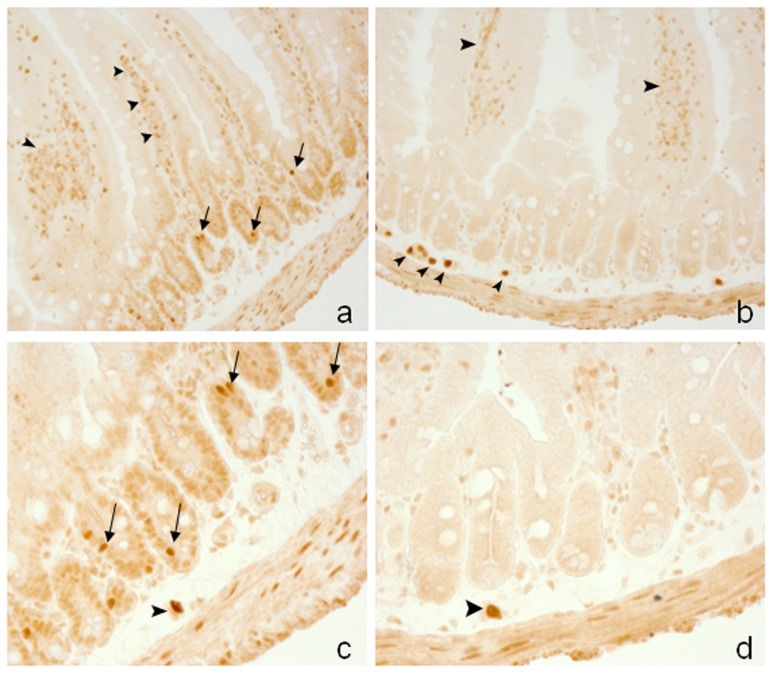
Gene targeting of *Ldb1* in the *villin-cre Ldb1^Δ/Δ^* mutant intestine. No defects in cell morphology of either villous or cryptal epithelium are observed in *villin-cre Ldb1^Δ/Δ^* mutant intestines (b, d) as compared to the *Ldb1^fl/fl^* control littermates (a, c). Most cells of the wild type crypt are expressed Ldb1 at low level. Strong positive signal is detected in a few cells of the crypt (arrows) as well as in the epithelial stroma (arrowheads) (x400). No Ldb1 protein is found in the crypts of the *villin-cre Ldb1^Δ/Δ^* mutant intestines, while stromal cells of the mutant are still expressed Ldb1 (arrowheads).

Expression of Ldb1 and Isl1 proteins were analyzed by immunohistochemistry with specific antibodies. Ldb1 was expressed at low level in the nuclei of most cells within the crypt, but some cells at the bottom of the crypt showed a much stronger signal (Figure 2b, 2c and Figure 3a, 3c). The number of cells prominently expressing Ldb1 was determined. Altogether, we found 214 Ldb1^high^ cells in 150 crypts analyzed. These cells were located at positions +3–+9, and the majority of Ldb1^high^ cells occupied positions +4–+7 (Figure 2j). There was no Ldb1 protein found in the villous epithelium (Figure 3). Many Ldb1-expressing cells were also detected in lamina propria where all cells are of mesenchymal origin (Figure 3).

Recently, the expression pattern of Ldb1 protein in small intestine also was reported by Dey-Guha et al. [33]. Although they used the same anti-Ldb1 antiserum as we did, the reported expression pattern was different. In contrast to our results, they showed high level of Ldb1 expression in both crypts and villi. One of the reasons for this discrepancy may have been the less stringent conditions used for immunostaining that could have resulted in a non-specific signal. In our experiments the effective antibody titer was carefully determined, and the IgG concentration of the pre-immune serum that served as a negative control was the same as that of specific antibodies (see Methods). Incubation of tissue sections of the *Ldb1^Δ/Δ^* mutant intestine with Ldb1 specific antibodies was applied as an additional negative control.

Next, we addressed the question of the origin of Ldb1-expressing cells in small intestine. In addition to the cells of endodermal origin, small intestine is normally populated by different types of lymphocytes and macrophages [4]. Some of the immune cells may intercalate into the layer of intestinal epithelium. Some cells of lymphoid lineage have been reported to produce the Ldb1 protein [34]. In order to discriminate between intestinal epithelial cells and leucocytes that might express Ldb1, we used antibodies for a common leucocyte marker CD45. There were numerous CD45 positive cells in the stroma of the intestine, and some cells intercalated the intestinal epithelium (data not shown). Double immunostaining with Ldb1 and CD45 antibodies revealed no Ldb1-expressing cells among the population of CD45 positive leukocytes in the crypt (data not shown). Thus, the intraepithelial lymphocytes did not express Ldb1. To confirm the epithelial origin of Ldb1 producing cells, we probed gut tissue sections with antibodies for cytokeratin, an epithelial cell marker. These experiments showed that Ldb1-expressing cells in the crypt are also marked by cytokeratin (Figure S1a–c), suggesting the endodermal origin of these cells.

Cells expressing Isl1 protein were found only in the crypt compartment (Figure2e and f). However, in contrast to Ldb1^high^ cells, only few Isl1 producing cells (29 Isl1+ cells versus 214 Ldb1^high^ cells within 150 crypts) were found in the crypt based on analysis of serial sections (Figure 2e and f). Isl1+ cells occupied position +3/+4 at the base of the crypt. No Isl1 positive cells were observed in the villous epithelium or in lamina propria. Co-immunostaining with Ldb1-specific antibodies showed that both factors were co-expressed in the same cells of the crypt (Figure 2g–i). All Isl1 positive cells also expressed Ldb1 at high level. However, some cells that prominently expressed Ldb1 were negative for Isl1 (Figure 2i).

Altogether, our analysis of Ldb1 and Isl1 expression pattern showed that cells expressing both factors are localized at the bottom of the crypt in the niche of intestinal stem cells and early precursors. The majority of crypt cells represent a population of intensively proliferating stem cells and early precursors. The descendants of these cells migrate first to TA (transit amplifying) zone and then to the villi to replenish the villous epithelium and other intestinal lineages. There is also a population of quiescent slowly cycling intestinal stem cells [35]. In order to differentiate between these two cell populations in the crypt, we analyzed the proliferation potential of Ldb1/Isl1 expressing cells.

### Ldb1/Isl1 expressing cells are quiescent

To evaluate the proliferative activity of the cells expressing both Ldb1 and Isl1 we used a number of cell proliferation markers. Ki67 is known as a general proliferation marker, BrdU indicates cells at S-phase, and phosphorylated histone H3 (PH3) is a cell marker of mitosis.

**Figure 4:**
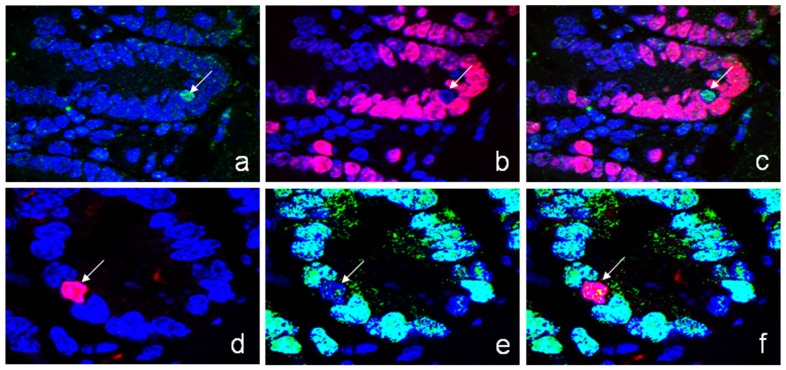
Cell proliferation in the crypt. Cell proliferation is evaluated with antibodies for Ki-67 antigen. Cells expressing Ldb1 (a–c) and Isl1 (d–f) are negatively stained by Ki-67 proliferation marker. **a**. Ldb1-expressing cells (green); **b**. Ki-67-expressing proliferative cells (red); **c**. Merge of **a** and **b** (x1000). **d**. Isl1-expressing cells (red); **e**. Ki-67-expressing cells (green); **f**. Merge of **d** and **e** (x1000). Arrows point on cells expressing either Ldb1 (a, b, c) or Isl1 (d, e, f). Nuclei are counterstained with DAPI (blue).

While many cells in the crypt were positively stained for Ki67 as expected, there was no expression of Ki67 in Ldb1-expressing cells (Figure 4a–c). Isl1-expressing cells similarly lacked the Ki67 protein (Figure 4d–f). Furthermore, no PH3 histone was detected in Ldb1/Isl1 positive cells (Figure S2a and S2b). Thus, it is unlikely that these cells belong to the group of intensively proliferating stem cells/progenitors; but, they might be slowly cycling stem cells or their direct descendants - so called “daughter” cells.

**Figure 5:**
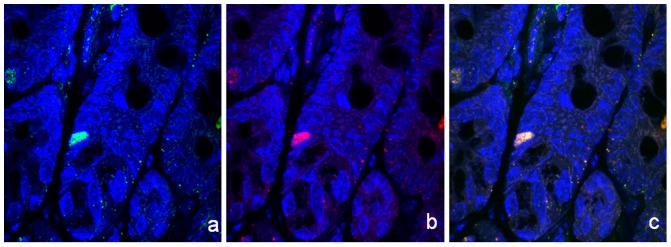
Long term BrdU retention. Cells of the gut were labeled with BrdU. Retention of the signal is analyzed on the 20^th^ day after BrdU injection. **a, b, c**. Co-localization of Ldb1 (red) and BrdU (green) in the intestinal cells. **a**. BrdU, **b**. Ldb1, **c**. Merge of **a** and **b** (x1000). Nuclei are counterstained with DAPI (blue).

To test this assumption, we labeled nuclei of the crypt cells with BrdU and analyzed retention of the incorporated BrdU in a long-term labeling experiment. Following 20 days after initial BrdU injection, only few cells in the crypt were able to retain the label (35 BrdU-positive cells, n = 150, where n is a number of crypts analyzed), while the signal in intensively proliferating cells from the TA zone was diluted out (Figure 5). Double staining with Ldb1 antibodies revealed 15 Ldb1^high^ cells (n = 150) within the population of BrdU label retaining cells (Figure 5). BrdU+/Ldb1^high^ cells were located at position +3/+4.

There are several groups of cells within crypt that might retain BrdU for a long time period and show low proliferation level. First, there are the terminally differentiated cells, namely Paneth, Goblet cells or enteroendocrine cells. Ldb1/Isl1 positive cells unlikely are Goblet cells which can be easily distinguished by their typical morphology. We also were unable to identify these cells with Paneth cells, because they are well characterized by their granularity, whereas Ldb1/Isl1-expressing cells are not granulated. Similarly, Ldb1/Isl1 positive cells do not represent a subtype of enteroendocrine cell lineage that is marked by Chromogranin A [36], [37], because these cells are not expressing Chromogranin A (Figure S1d). Second, cells undergoing apoptosis also should retain BrdU and lack of Ki-67 expression [38]. To test this possibility we co-immunostained gut tissue sections with antibodies for Isl1 and antibodies for Cleaved Caspase3, the marker of apoptosis. Isl1 positive cells were not stained positive with the Caspase3 antibodies (Figure S2c), suggesting the Isl1/Ldb1-expressing cells are not apoptotic cells.

Thus Isl1^+^/Ldb1^high^-expressing cells belong to neither any known, terminally differentiated cell lineages populating the crypt nor are they associated with the group of intensively proliferating precursors. Most likely these cells represent a population of quiescent LRC stem cells and/or their early progenitors. Recent lineage tracing studies identified LRC cells as progenitors of Paneth and enteroendocrine cell lineages [35]. Importantly, these cells undergo intensive proliferation and are capable of the clonogenic growth upon intestinal injury [31], [35]. Thus, LRC cells may represent a reserve pool of stem cells that are activated in regenerating gut tissue.

### Ldb1 has no specific role in small gut endoderm

In order to evaluate the biological role of *Ldb1* in small intestine we generated mice where *Ldb1* gene was targeted conditionally in the gut endoderm. To specifically delete *Ldb1* gene in the intestinal endoderm, mice carrying *Ldb1^fl/fl^* floxed allele were intercrossed with *villin-cre* mice [39]. The villin promoter is active at E14 stage of gestation, and the cre recombinase efficiently removes the target gene in all gut endodermal cells [39]. Furthermore, *villin-cre* remains active throughout gestation and in the adult gut tissue [39].

*Villin-cre Ldb1^fl/fl^* mice were born in the expected Mendelian ratio and remained viable and healthy throughout their lifetime. Mice were active and gained weight in a manner similar to their wild type littermates. Gene deletion in gut tissue was confirmed by immunohistochemistry with Ldb1 specific antibody (Figure 3). Morphological and histological analysis revealed no significant differences in the structure and patterning of small intestine between mutant and wild type mice (Figure 3). The size and number of villi and crypts was similar in both groups of animals (data not shown). In order to better characterize the mutation, we quantitatively assessed the cell number in specific lineages within different compartments of the small intestine. There was no substantial difference in the number of Goblet and Paneth cells between mutant and wild type intestines (data not shown). Therefore, the role of Ldb1 in small gut endoderm is either dispensable or redundant since *Ldb2* is expressed at relatively high level (Figure 1) and might partially compensate the function of *Ldb1* in mutant cells.

The phenotype described in this study is different of that recently published by Dey-Ghua et al. [33]. These authors used tamoxifen-inducible ROSA26-cre recombinase to delete the floxed *Ldb1* allele. They reported severe defects in the small intestine tissue and high lethality rate for the mutant mice. Ldb1 protein is expressed in many organs and tissues, and targeted deletion of its gene in mice results in an embryonic lethality and a pleiotropic phenotype [40]. Given that ROSA26 promoter is expressed ubiquitously, it is conceivable the *Ldb1* gene could be targeted in multiple tissues and organs of the mutant mice. Thus, the intestinal phenotype observed by Dey-Guha et al. could be secondary to the primary defect induced by *Ldb1* ablation in other organs and tissues. Indeed, renal failure was reported in podocyte-specific *Ldb1* knock-out mice [41]. Also, the essential role of *Ldb1* was demonstrated recently for the maintenance of hemapoietic stem cells [34]. Furthermore, it was recently shown that the tamoxifen used to induce *cre* expression in experiments of Dey-Guha et al. was able to promote apoptosis in the crypt compartment [38]. Thus, it is remains unclear whether reported high level of cell death is a consequence of the *Ldb1* gene loss or the result of the side effect of tamoxifen.

In contrast to the work of Dey-Guha et al., our data revealed no specific function for *Ldb1* in gut endoderm. However, we cannot exclude a possible gene redundancy. Also, the *Ldb1* role may be important for the intestinal stroma. Further experiments should be performed to specifically target the *Ldb1* gene in gut mesoderm. Since analysis of the mutant phenotype was conducted under normal physiological conditions, it would be useful for future studies to focus on phenotypes induced by physiological stress.

In this study we described the expression pattern of LIM homeodomain transcription factors and Ldb1/Ldb2 co-factors in the small intestine. We found that the cells expressing both Ldb1 and Isl1 proteins are present only in the crypt compartment. These cells have been identified as a population of quiescent slow cycling cells. It will be interesting to determine if Isl1 and Ldb1^high^ could serve as markers for the LRC intestinal stem cell population. This question could be addressed in future studies by lineage tracing experiments. This work highlights the importance of new marker identification to the study of complexity of intestinal stem cells homeostasis and cell fate determination.

## Supporting Information

Figure S1 **Characterization of Ldb1-expressing cells in the crypt.** **a,b,c.** Ldb1-expressing cells (green) in the crypt are expressed also cytokeratin (red). **c**. Merge of **a** and **b** (x1000). **d.** Cells positively stained for Isl1 (green) do not express the enterocyte marker Chromogranin A (red) (x1000). Arrows indicate Ldb1-expressing cells (a, c).
(TIF)

Figure S2 **Mitosis and cell death.** Mitosis (a, b). Cells expressing either Ldb1 (green in **a**) or Isl1 (green in **b**) are not expressing PH3 histone mitosis marker (red) (x1000). Arrows indicate Ldb1-expressing cells (a) and Isl1-expressing cells (b). Cell death (c). No Cleaved Caspase-3 (green) expression is found in the cells positively stained for Isl1 (red) (x1000).
(TIF)
