# Supplementary figures and images for: Islet1 and Its Co-Factor Ldb1 Are Expressed in Quiescent Cells of Mouse Intestinal Epithelium
Source: PLoS One. 2014 Apr 22;9(4):e95256. doi: 10.1371/journal.pone.0095256 (PMC3995853; doi:10.1371/journal.pone.0095256)

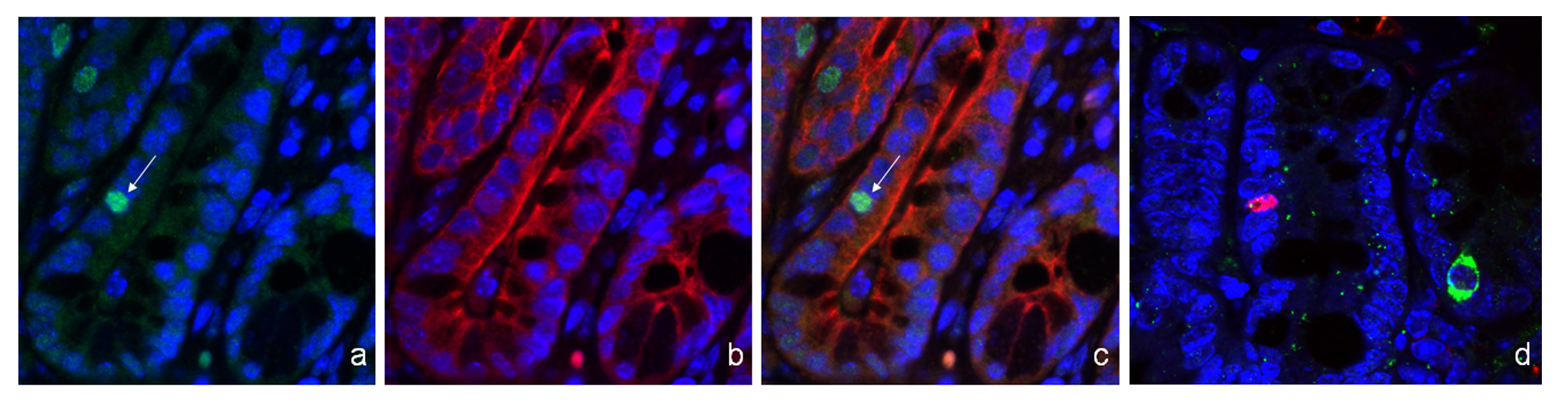

Supplement: Figure S1 — Characterization of Ldb1-expressing cells in the crypt. a,b,c. Ldb1-expressing cells (green) in the crypt are expressed also cytokeratin (red). c. Merge of a and b (x1000). d. Cells positively stained for Isl1 (green) do not express the enterocyte marker Chromogranin A (red) (x1000). Arrows indicate Ldb1-expressing cells (a, c). (TIF) [file pone.0095256.s001.tif]

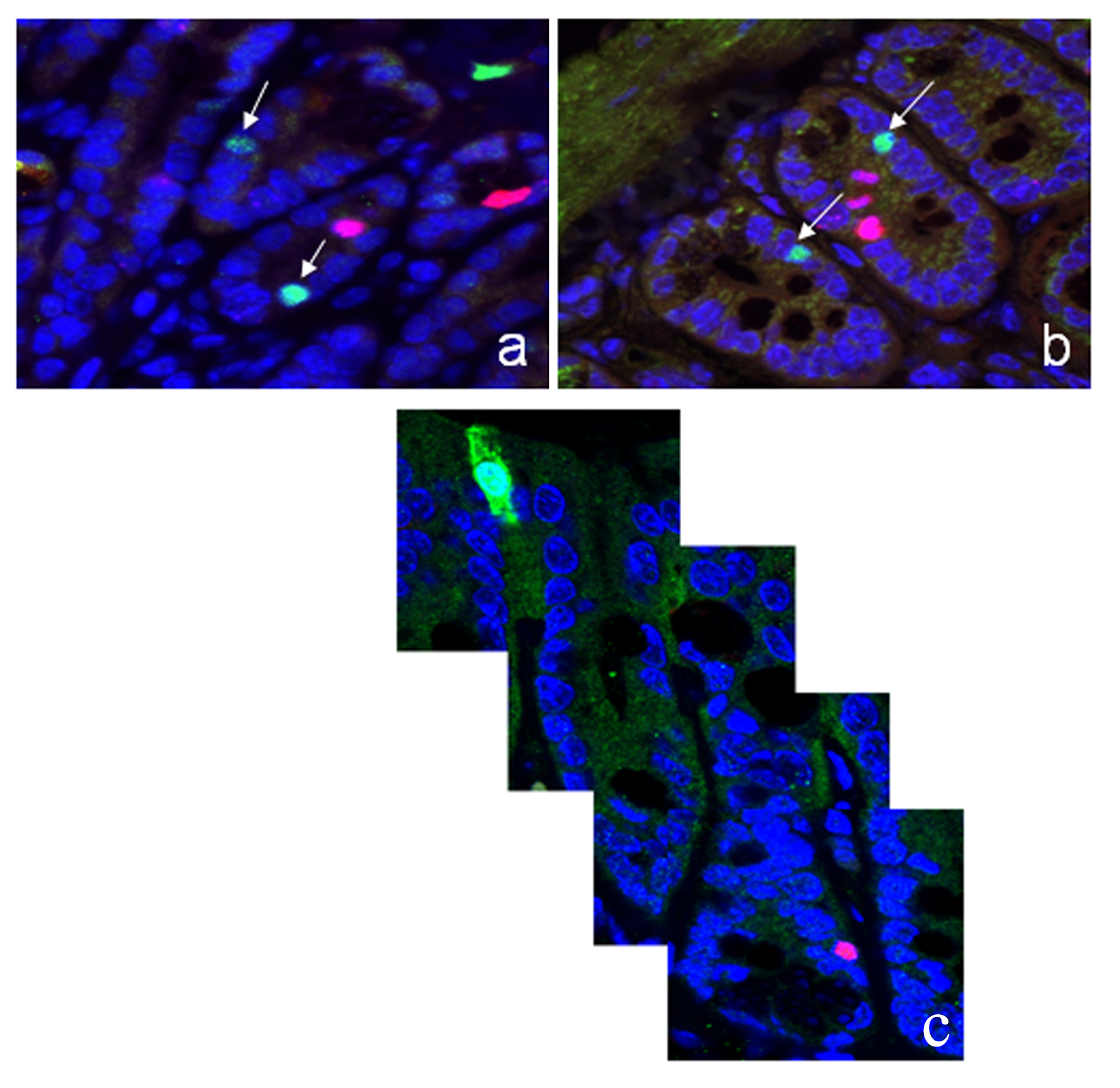

Supplement: Figure S2 — Mitosis and cell death. Mitosis (a, b). Cells expressing either Ldb1 (green in a) or Isl1 (green in b) are not expressing PH3 histone mitosis marker (red) (x1000). Arrows indicate Ldb1-expressing cells (a) and Isl1-expressing cells (b). Cell death (c). No Cleaved Caspase-3 (green) expression is found in the cells positively stained for Isl1 (red) (x1000). (TIF) [file pone.0095256.s002.tif]
